# Risk Factors for Visceral Leishmaniasis Relapse in Immunocompetent Patients following Treatment with 20 mg/kg Liposomal Amphotericin B (Ambisome) in Bihar, India

**DOI:** 10.1371/journal.pntd.0002536

**Published:** 2014-01-02

**Authors:** Sakib Burza, Prabhat K. Sinha, Raman Mahajan, María Angeles Lima, Gaurab Mitra, Neena Verma, Manica Balsegaram, Pradeep Das

**Affiliations:** 1 Médecins Sans Frontières, New Delhi, India; 2 Rajendra Memorial Research Institute of Medical Sciences, Patna, Bihar, India; 3 Médecins Sans Frontières, Barcelona, Spain; 4 ACCESS Campaign, Geneva, Switzerland; Emory University, United States of America

## Abstract

**Background:**

A proportion of all immunocompetent patients treated for visceral leishmaniasis (VL) are known to relapse; however, the risk factors for relapse are not well understood. With the support of the Rajendra Memorial Research Institute (RMRI), Médecins Sans Frontières (MSF) implemented a program in Bihar, India, using intravenous liposomal amphotericin B (Ambisome) as a first-line treatment for VL. The aim of this study was to identify risk factors for VL relapse by examining the characteristics of immunocompetent patients who relapsed following this regimen.

**Methods and Principal Findings:**

This is an observational retrospective cohort study of all VL patients treated by the MSF program from July 2007 to August 2012. Intravenous Ambisome was administered to 8749 patients with VL in four doses of 5 mg/kg (for a total dose of 20 mg/kg) over 4–10 days, depending on the severity of disease. Out of 8588 patients not known to be HIV-positive, 8537 (99.4%) were discharged as initial cures, 24 (0.3%) defaulted, and 27 (0.3%) died during or immediately after treatment. In total, 1.4% (n = 119) of the initial cured patients re-attended the programme with parasitologically confirmed VL relapse, with a median time to relapse of 10.1 months. Male sex, age <5 years and ≥45 years, a decrease in spleen size at time of discharge of ≤0.5 cm/day, and a shorter duration of symptoms prior to seeking treatment were significantly associated with relapse. Spleen size at admission, hemoglobin level, nutritional status, and previous history of relapse were not associated with relapse.

**Conclusions:**

This is the largest cohort of VL patients treated with Ambisome worldwide. The risk factors for relapse included male sex, age <5 and ≥45 years, a smaller decrease in splenomegaly at discharge, and a shorter duration of symptoms prior to seeking treatment. The majority of relapses in this cohort occurred 6–12 months following treatment, suggesting that a 1-year follow-up is appropriate in future studies.

## Author Summary

Visceral leishmaniasis (VL), also known as Kala azar, accounts for the second-highest burden of parasitic disease worldwide. Fifty percent of VL cases worldwide occur in India, and up to 90% of these in the state of Bihar. Up to 10% of VL patients relapse within 6 months of treatment, particularly if co-infected with HIV. Limited data are available regarding relapse in patients with intact immune systems. Between 2007–2012, with support of the Rajendra Memorial Research Institute(RMRI), Médecins Sans Frontières (MSF) treated VL patients in Bihar with 20 mg/kg liposomal amphotericin B (Ambisome). Here we identify risk factors for relapse in immunocompetent patients by comparing the demographics and clinical characteristics of 119 patients testing negative for HIV who experienced parasitologically-confirmed VL relapse against those of the remaining 8418 patients not known to have relapsed. Male sex, age <5 years and ≥45 years, shorter duration of symptoms prior to seeking treatment, and a smaller reduction in spleen size by time of discharge were all risk factors for relapse. The majority of relapses occurred 6–12 months post-treatment with Ambisome. The conventional period of follow-up is 6 months. Considering the aim of elimination in the Indian subcontinent, this data suggests that 1-year follow-up is necessary.

## Introduction

Visceral leishmaniasis (VL) is a neglected tropical disease that results in the loss of an estimated 1 million disability-adjusted life years annually in South East Asia [1]; it is typically fatal if untreated. VL predominantly affects the poorest strata of society and those with limited access to care [2]. The incidence is estimated to be between 146,700 and 282,800 cases per year [3]. Fifty percent of VL cases worldwide occur in India, and up to 90% of these in the state of Bihar.

Although complete parasite clearance is rarely achieved, it is thought that patients with competent immune systems who are successfully treated develop an effective lifelong cellular immune response that suppresses residual parasite growth [4]. High relapse rates in HIV-positive patients have been previously described [5], [6]. However, in nearly all studies assessing treatment effectiveness, a proportion of immunocompetent patients relapse following treatment despite negative end-of-treatment test-of-cure results. Typically, these relapses occur within 6 months of initial treatment with later recurrence considered rare [7]. Very little is known regarding the characteristics of immunocompetent patients with VL who relapse [8], [9], particularly in the Indian context.

The aim of this observational retrospective cohort study was to identify risk factors for relapse in immunocompetent patients who had been treated with 20 mg/kg Ambisome for their primary episode of VL. With the permission of the State Health Society of Bihar and the support of the specialist VL research institute the Rajendra Memorial Research Institute (RMRI), Médecins Sans Frontières (MSF) has been treating patients with VL in Vaishali district since 2007, in coordination with the National Vector Borne Disease Control Programme of India. Between July 2007 and August 2012, a total of 8749 patients diagnosed with VL were treated with intravenous 20 mg/kg liposomal amphotericin B (Ambisome; Gilead Pharmaceuticals, Foster City, CA, USA). This regimen has been shown to have a 6-month cure rate of 98% in the Indian context [10].

Ambisome is a brand name for Liposomal Amphotericin B. There are a number of preparations of Liposomal amphotericin B available on the market; however due to the lack of standard and widely applicable regulations or guidance for liposomal technology, it is important that this specific preparation be named. At time of publication, none of the rival preparations have undergone peer reviewed non-inferiority studies against Ambisome nor received stringent regulatory approval for use in VL. It is for this reason that MSF and the WHO currently only use Ambisome rather than other preparations. However it is urgent that clear regulatory guidelines for endemic countries be established by a normative setting organisation like the WHO and other existing formulations be formally evaluated [11].

Using the data routinely collected from the MSF program, we determined the demographic and clinical characteristics of 119 immunocompetent patients who presented back to the program with parasitologically confirmed VL relapse. We then identified possible risk factors for relapse by comparing these patients to the 8418 patients who were discharged as cured and were not known to have relapsed.

## Methods

MSF developed an integrated program within the existing healthcare facilities in Vaishali district, Bihar, that utilised the district hospital for inpatient care and five rural primary health centers for ambulatory treatment in the community. Patients diagnosed with VL at the primary health center level who were aged <2 years or >55 years, pregnant or lactating, severely malnourished, had a hemoglobin (Hb) level of <5 g/dL, or had a history of relapse or HIV infection were referred to the district hospital for further assessment.

All patients with a history consistent with VL (fever for >2 weeks and splenomegaly) had their diagnosis confirmed using rK39 rapid diagnostic tests (Diamed-IT LEISH; DiaMed AG, Cressier, Switzerland). Patients presenting with a previous history of treatment for VL or with continued suspicion of VL despite negative diagnostic tests were admitted at the district level hospital or referred to a higher center (RMRI) for parasitological confirmation.

Upon diagnosis of VL, the basic demographics of the patients were recorded: clinical history, Hb level, malaria rapid diagnostic test result, and nutritional status. Also recorded was ‘caste’, a form of social stratification used in India; the categories used in the study were: scheduled caste/tribe, other backward class and general category. Other backward class is a collective term used by the government of India for castes that are educationally and socially disadvantaged. Scheduled caste/tribe are terms used for two groups of historically disadvantaged people recognized in the Constitution of India. These three groups combined account for approximately 60% of India's population. The general category is for those who do not fit into the other categories and are, therefore, not considered disadvantaged.

Initially, HIV tests were offered at the hospital level to those who were deemed high-risk (e.g. patients who presented with VL relapse or those who had a history suggestive of higher risk, such as being a migrant worker). This was changed in March 2011 when all inpatients aged >14 years were offered HIV testing. All women of childbearing age were given urinary pregnancy tests.

Patients were treated with four doses of 5 mg/kg Ambisome over 4–10 days, depending on the clinical severity. Initially, all patients with VL were treated on days 0, 1, 4, and 9. However, due to the increasing number of patients and the limited capacity of the hospital, the treatment duration was reduced to 4 consecutive days at the hospital level for all clinically stable patients. Patients given ambulatory treatment at the primary health centers and clinically severe patients admitted to the hospital continued to receive the 7–10 day regimen.

An initial cure was defined as improvement of symptoms, cessation of fever, and reduction of spleen size at time of discharge. Considering the risks associated with splenic puncture, a test-of-cure was performed only on patients with suspected treatment failure, particularly as an earlier field study using this regimen in the same setting showed a 6 month final cure rate of >98% [10]. Fortunately, no patients during the 5-year study period were suspected of treatment failure.

**Figure 1:**
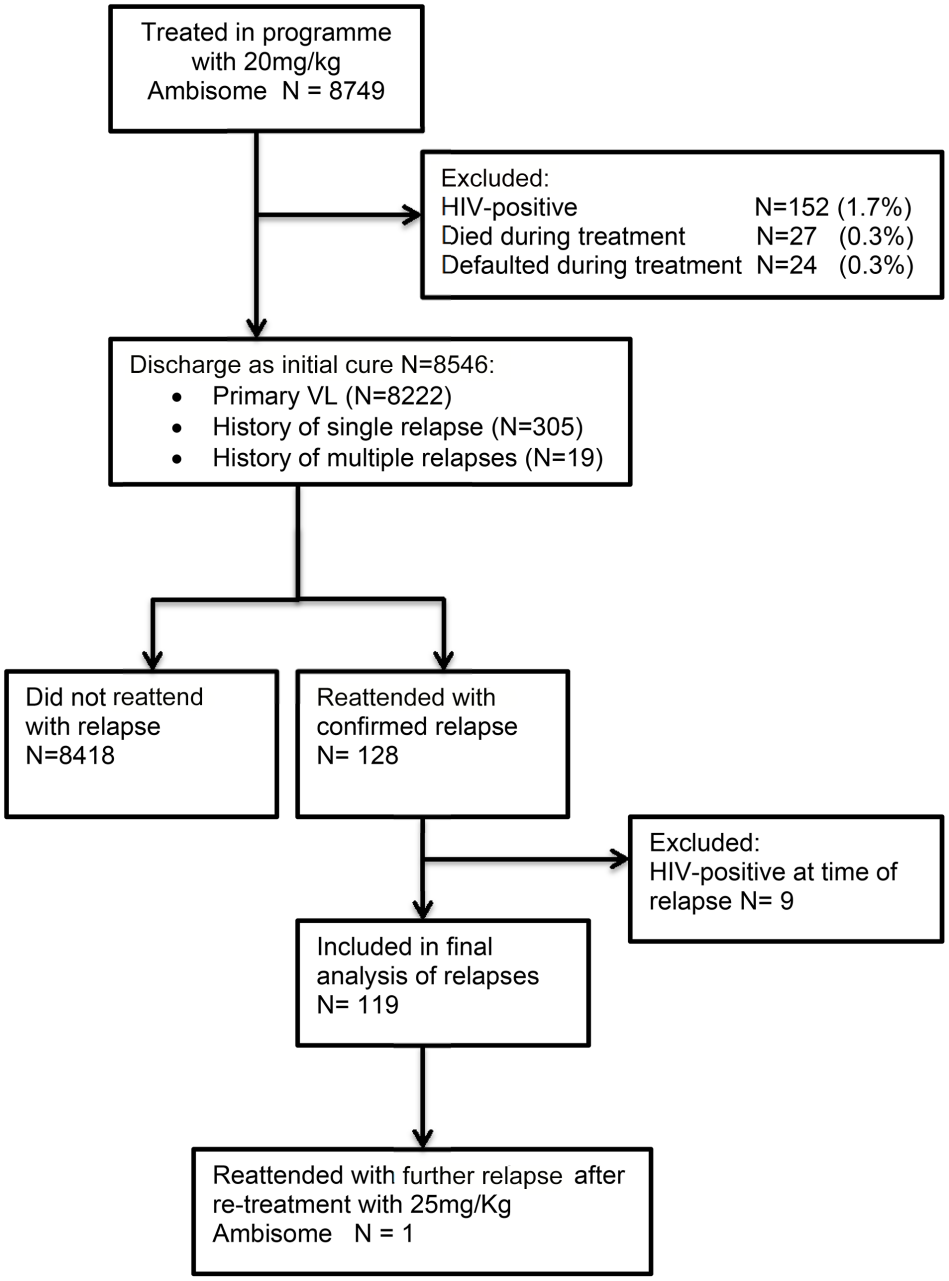
Flowchart of the selection procedure for the patient record analysis.

All patients were provided with health education regarding VL and the possibility of relapse, and were advised to return to the MSF-supported district hospital if symptoms recurred. Patients who returned to the program with suspected relapse were matched with their original records, and parasitological confirmation was sought. If splenomegaly was present, splenic aspiration was performed. In cases without splenomegaly, or where splenic aspiration was contraindicated (e.g. low platelet count), bone marrow aspiration was performed. All patients with confirmed VL relapses and an unknown HIV status were offered an HIV test. All patients testing positive for HIV were excluded from the analysis (Figure 1).

Patients with a parasitologically confirmed relapse after treatment with the 20 mg/kg Ambisome regimen were then treated with 25 mg/kg Ambisome in 5 successive doses at the district hospital level.

All data were entered into a standard Microsoft Excel database; double data-entry was not performed. Database cleaning was performed at regular intervals, including checks for inconsistencies and verification by comparing with the source document, when available. An epidemiologist ensured that the database was well-maintained and made regular quality audits of data transfer. World Health Organization (WHO) Anthro and Anthro Plus software (Geneva, Switzerland) were used to calculate the weight-for-height Z-score for children aged <5 years and the BMI-for-age Z-score for children aged ≥5–19 years. A retrospective analysis of all routinely collected program data was then conducted using SPSS version 19 (IBM, Chicago, IL, USA).

The variables considered during the analysis included: age, sex, Hb level, spleen size at admission, decrease in spleen size at the time of discharge, nutritional status, time to presentation (duration of symptoms prior to seeking treatment), location of treatment (inpatient vs ambulatory), duration of treatment with Ambisome (4 vs. 7–10 days), season at time of treatment (November–February vs March–June vs July–October), caste, and history of previous relapse. Due to the differences in treatment durations, the decrease in spleen size at time of discharge variable was standardised by dividing the change in spleen size by the number of days that the patient was admitted, yielding an average value of cm change-per-day of admission (≤0.5 vs. >0.5 cm/day).

### Ethics statement

This analysis met the Médecins Sans Frontières Institutional Ethics Review Committee's criteria for a study involving the analysis of routinely collected program data. Although a new treatment in the Indian setting, the programme utilised a recognised treatment for VL and was run in coordination with the State Health Society through a memorandum of understanding, which is the usual procedure for NGOs operating in this context. All electronic data were analysed anonymously.

## Results

Over the 5-year period from July 2007 to December 2012, 8749 patients with VL were treated in the MSF program (Figure 1). Demographic data for the cured cohort and patients who experienced VL relapses are in Table 1. The overall program mortality and default from treatment rates were very low (0.3% each). Overall, disproportionately more male patients (56.6%) than female patients were treated; only 52.9% of the population in Bihar is male [12]. Most patients were from lower castes, and nearly half were aged <15 years. Most patients had no previous history of VL, and the monthly distribution of admissions reflected the known seasonality of VL in Bihar.

**Table 1:** Association between demographic factors and VL relapse.

Risk factor	Cured cohort	Relapse cohort	Unadjusted odds ratio (95% CI)	p
Sex (n = 8537)	**8418**	**119**		
Male	4745 (56.4)	83 (69.7)	1.8 (1.2–2.6)	0.003
Female	3673 (43.6)	36 (30.3)	-	-
Caste^a^ (n = 8486)	**8368**	**118**		
Scheduled caste	2453 (29.3)	32 (27.1)	1.4 (0.7–2.7)	0.32
Other backward class	4624 (55.3)	74 (62.7)	1.7 (0.9–3.2)	0.08
General Category	1291 (15.4)	12 (10.2)	-	-
Living in MSF-supported blocks (n = 8537)	**8418**	**119**		
No	4297 (51.0)	56 (47.1)	0.9 (0.6–1.2)	0.39
Yes	4121 (49.0)	63 (52.9)	-	-
Previous relapse (n = 8537)	**8418**	**119**		
Multiple	18 (0.2)	1 (0.8)	3.9 (0.1–25.4)	0.23^c^
Single	299 (3.6)	4 (3.4)	0.95 (0.3–2.5)	1.00^c^
No	8101 (96.2)	114 (95.8)	-	-
Age, years (n = 8537)	**8418**	**119**		
<5	574 (6.8)	17 (14.3)	3.6 (1.8–7.1)	<0.001
5 to <15	3229 (38.4)	43 (36.1)	1.6 (0.9–2.9)	0.10
15 to <30	1935 (23.0)	16 (13.4)	-	-
30 to <45	1490 (17.7)	21 (17.6)	1.7 (0.9–3.3)	0.11
≥45	1190 (14.1)	22 (18.5)	2.2 (1.2–4.3)	0.013
Treatment location (n = 8537)	**8418**	**119**		
Treatment camps (ambulatory)	162 (1.9)	0	NA	-
Primary health center (ambulatory)	1372 (16.3)	13 (10.9)	0.6 (0.3–1.1)	0.10
Hospital (inpatient)	6884 (81.8)	106 (89.1)	-	-
Season of treatment (n = 8537)	**8418**	**119**		
March–June	3566 (42.4)	53 (44.5)	1.0 (0.7–1.6)	0.84
July–October	2527 (30.0)	33 (27.7)	0.9 (0.6–1.5)	0.74
November–February	2325 (27.6)	33 (27.7)	-	-
Year of treatment^a^^,^ b (n = 7471)	**7359**	**112**		
2011	1692 (23.0)	31 (27.7)	1.4 (0.8–2.2)	0.22
2010	1614 (21.9)	25 (22.3)	1.1 (0.7–1.9)	0.61
2009	1468 (19.9)	21 (18.8)	1.1 (0.6–1.8)	0.84
2007–2008	2585 (35.1)	35 (31.3)	-	-

^a^ The remaining data were missing or incorrectly coded in the database.

^b^ The data from years 2007 and 2008 were combined, as operations began mid-2007. Patients treated in 2012 were excluded, as there would be limited time for relapse to present.

^c^ Fisher's Exact test.

**Figure 2:**
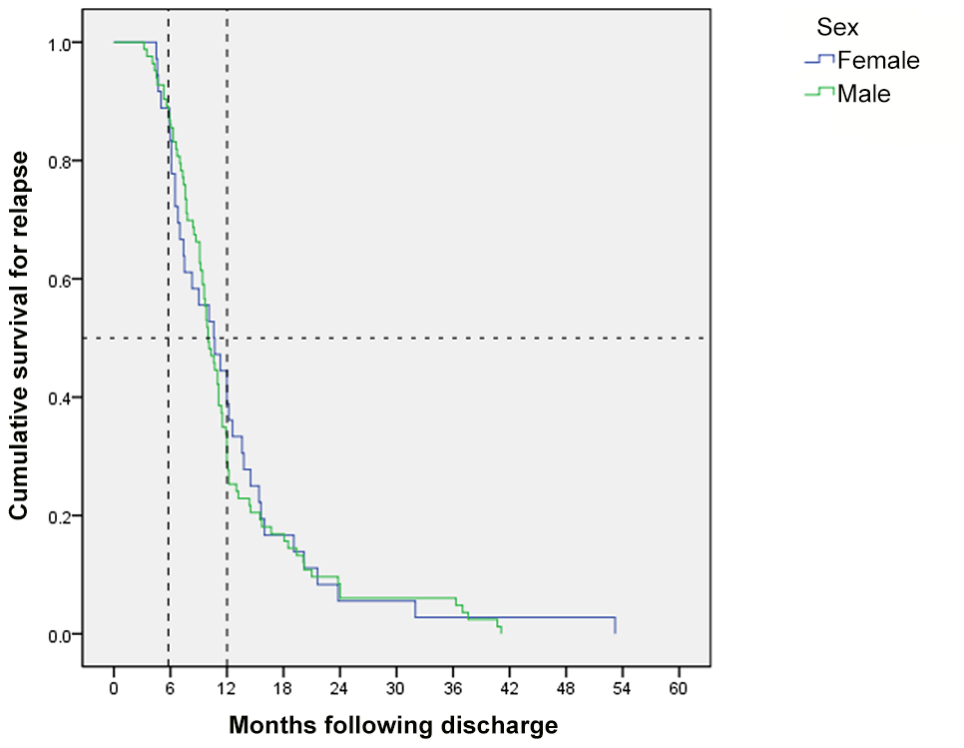
Uncensored Kaplan–Meier graph showing time to relapse stratified by sex (n = 119). *Log rank (Mantel-Cox) p = 0.776.

Patients who died during treatment, defaulted, or were HIV-positive were excluded from the final analysis (Figure 1). A total of 119 patients presented with parasitologically confirmed VL relapse following initial discharge. The median (interquartile range) time to relapse from initial treatment was 10.1 (7.1–13.8) months. Of the 119 patients who relapsed: 18 (15.1%) relapsed within 6 months of treatment; 63 (52.9%) within 6–12 months; and 38 (31.9%) >12 months after treatment. There was no significant difference in the meantime to relapse between the sexes (log rank Mantel-Cox p = 0.776) (Figure 2).

### Demographic risk factors

Male patients had higher odds of relapsing (unadjusted odds ratio [uOR] 1.8; 95% CI 1.2–2.6) compared with female patients. Patients aged <5 years (uOR 3.6; 95% CI 1.8–7.2) and ≥45 years (uOR 2.2; 95% CI 1.2–1.4) were more likely to relapse than patients aged ≥15 to <30 years. Factors not associated with relapse in the univariate analysis (p>0.05) included: caste; living in an area where MSF was conducting information, education, and communication activities as well as supporting the primary health center; a history of previous relapse; location of treatment administration (treatment camp or primary health center/hospital); and season or year of treatment.

### Clinical risk factors

According to the univariate analysis, patients reporting a shorter duration of symptoms prior to seeking treatment had higher odds of relapsing. Compared with the baseline group of patients who received treatment ≤4 weeks after developing symptoms (also known as time to presentation), the odds of relapse progressively decreased as the duration of symptoms prior to seeking treatment increased, from 0.6 (95% CI 0.4–0.97) for those presenting >4 to ≤8 weeks after symptoms occurred to 0.4 (95% CI 0.2–0.8) for those presenting >8 weeks after symptoms occurred. Additionally, patients who exhibited a decrease in spleen size of ≤0.5 cm/day by the time of discharge appeared to have higher odds of relapse (1.7; 95% CI 1.1–2.5) compared with those who exhibited a decrease in spleen size of >0.5 cm/day. No other clinical factors were significantly associated with risk of relapse (Table 2). Notably, nutritional status, spleen size and Hb level upon admission, and duration of treatment were not predictive of relapse.

**Table 2:** Association between clinical features and relapse of VL.

Risk factor^a^	Cured cohort	Relapse cohort	Unadjusted odds ratio (95% CI)	p
Nutritional status (n = 7055)	**6949**	**106**		
Severe acute malnutrition	1233 (17.7)	19 (17.9)	1.1 (0.6–1.8)	0.73
Moderate acute malnutrition	1587 (22.8)	29 (27.4)	1.3 (0.8–2.0)	0.25
Normal	4129 (59.4)	58 (54.7)	-	-
Duration of symptoms prior to seeking treatment, weeks (n = 8533)	**8414**	**119**		
≤4	4909 (58.3)	86 (72.3)	-	-
>4 to ≤8	2070 (24.6)	22 (18.5)	0.6 (0.4–0.97)	0.036
>8	1435 (17.1)	11 (9.2)	0.4 (0.2–0.8)	0.008
Spleen size at admission, cm (n = 8534)	**8415**	**119**		
<3	1229 (14.6)	21 (17.6)	-	-
3–6	4306 (51.2)	63 (52.9)	0.9 (0.5–1.4)	0.54
>6	2880 (34.2)	35 (29.4)	0.7 (0.4–1.2)	0.22
Decrease in spleen size at discharge, cm/day (n = 8513)	**8394**	**119**		
≤0.5	4995 (59.5)	85 (71.4)	1.7 (1.1–2.5)	0.008
>0.5	3399 (40.5)	34 (28.6)	-	-
Hb level, g/dL (n = 8512)	**8393**	**119**		
<6	1082 (12.9)	12 (10.1)	0.7 (0.3–1.3)	0.22
≥6–≤8	2782 (33.1)	43 (36.1)	0.9 (0.6–1.5)	0.72
>8–≤10	2746 (32.7)	34 (28.6)	0.7 (0.4–1.2)	0.22
>10	1783 (21.2)	30 (25.2)	-	-
Duration of treatment, days (n = 8524)	**8405**	**119**		
4	1709 (20.3)	21 (17.6)	0.8 (0.5–1.3)	0.47
7–10	6696 (79.7)	98 (82.4)	-	-

^a^ Where patient numbers are <8537, the remaining data were missing or incorrectly coded in the database.

### Multivariate regression model of risk factors for relapse

A multivariate logistic regression model was developed for those variables that were shown to be significant by univariate analysis (p<0.05) (Table 3). Variables that were justified a priori or were associated with relapse in other studies [8], [9] were also included in the multivariate analysis. These additional variables, which included nutritional status and spleen size and Hb levels upon admission, were added step-wise to the model. However, the variables associated with relapse as determined by the univariate analysis remained significant in the multivariate analysis, and those that were non-significant in the univariate analysis did not attain significance in the multivariate analysis.

**Table 3:** Multivariate analysis of risk factors for relapse.

Risk factor	Adjusted odds ratio	95% CI	p
Sex			
Male	1.74	(1.17–2.59)	**0.006**
Female	1	-	
Duration of illness prior to seeking treatment, weeks			
>8	0.45	(0.24–0.85)	**0.013**
>4 to ≤8	0.62	(0.38–0.99)	**0.045**
≤4	1	-	
Age, years			
<5	3.44	(1.72–6.88)	**<0.001**
5 to <15	1.51	(0.85–2.71)	0.162
15 to <30	1	-	
30 to <45	1.70	(0.88–3.26)	0.114
≥45	2.14	(1.12–4.11)	**0.022**
Decrease in spleen size at discharge, cm/day			
≤0.5	1.55	(1.03–2.32)	**0.035**
>0.5	1	-	

## Discussion

Debate regarding the role of reinfection rather than relapse in recurrence of VL is ongoing, however recent evidence supports the general consensus that relapse is more likely to be the cause [13]. Future studies investigating recurrence of VL will be strengthened by the assessment of different immunological parameters and pre- and post-relapse parasite fingerprinting in order to definitively answer this question.

### Risk factors for relapse

This cohort represents the largest number of patients with VL treated with Ambisome both worldwide and on the Indian subcontinent to date. Although based only in one district, this program has treated an estimated 5.8% of all reported VL cases in India between 2008 and 2011 [14]. The present study is also the only India-based study that specifically examines risk factors for and characteristics of relapse in immunocompetent patients, and describes the distribution of VL relapses >6 months after treatment. It is, therefore, of particular interest considering the move towards lower dose Ambisome as the first-line therapy for VL on the Indian subcontinent [7].

A strength of this study is the robust database that has been maintained throughout the program and has minimal missing data. A limitation is that all patients were not followed up prospectively to determine relapse status, and as such the identification of relapses depended on patients returning to the programme for assessment if their symptoms recurred. This may result in an underestimation of the number of relapses to the 20 mg/kg regimen.

There are limited data available regarding VL relapses in immunocompetent patients, and risk factors for relapse appear to vary from country to country. A retrospective study of 300 VL patients treated with meglutamine antimoniate in Georgia between 2002 and 2004 identified 21 cases of relapse. Among these cases, age <1 year, time to treatment of >90 days, and Hb levels of <6 g/dL were associated with relapse [8]. However, it is unclear whether these patients were tested for HIV. No association between relapse and spleen size nor sex was observed.

A more recent study examined patient characteristics and drug regimens associated with VL relapse in South Sudan between 1999 and 2007. The treatment records for 166 patients with VL who presented with relapses were compared with the treatment records for 7924 primary VL patients who did not re-attend with relapse [9]. This study found that larger spleen size upon admission and at the time of discharge were strongly associated with relapse, as was treatment with a short-course combination treatment (17 days sodium stibogluconate/paromomycin vs 30 days sodium stibogluconate). Age, sex, nutritional status, mobility, and treatment complications were not significantly associated with relapse. The main limitation was missing data, which resulted in the inclusion of only 26.7% (166/621) of the relapses in the analysis. Additionally, HIV testing was not performed for the relapse patients, although the authors considered this unlikely to be a factor for relapse in this group, as the estimated prevalence of co-infection was only 0.5%.

Our results suggest that age <5 and ≥45, male sex, a decrease in spleen size of ≤0.5 cm/day at discharge, and a short duration of symptoms prior to seeking treatment are risk factors for VL relapse in immunocompetent patients in India. Younger patients may be particularly susceptible to relapse due to the lack of a mature immune system [8]. The increased number of male patients presenting with relapse may be explained by the possibility of limited access to care for females. Indeed, in an analysis of the overall field outcomes of the MSF program in Bihar, the proportion of females admitted to the program progressively decreased in older age groups [Submitted to PLOS NTD].

We are unable to explain the strong inverse correlation that the present study revealed between the duration of symptoms prior to seeking treatment (time to presentation) and relapse. Indeed, this correlation is contrary to a priori knowledge, which would predict an association between a longer duration of illness and a more severe clinical presentation, and therefore more serious outcomes. However, other indicators of prolonged illness, such as low Hb level, poor nutritional status and increased splenomegaly at time of admission, were also not associated with relapse. It is possible that, in the Indian context, a rapid presentation to the healthcare provider could itself be an independent indicator of more severe illness or poorer immune status. This association needs to be correlated with other programmes' outcomes and warrants further investigation.

### Timing and characteristics of relapses

In India, the synthetic phospholipid derivative hexdecylphosphocholine (miltefosine) is currently recommended by the Indian National Programme as the first line treatment for VL. It is a 28-day oral treatment but its use is limited by teratogenicity, which restricts its use in pregnant and lactating women and requires 3–5 months of contraceptive cover for women of childbearing age [15]. Although miltefosine initially showed promising efficacy and tolerability, recent studies in India [16], [17] and Nepal [13] have demonstrated relapse rates in immunocompetent patients of between 6.8% to 10.8% at 6 months respectively, and up to 20.0% at 12 months in Nepal.

Over the past decade, several studies have examined liposomal preparations of amphotericin B. For doses of Ambisome ≥10 mg/kg, the efficacy at 6 months is >95% [11]. Following a pivotal phase III study published in 2010 [18], the WHO expert committee on leishmaniasis adopted a single 10 mg/kg dose regimen as the recommended first-line treatment for VL in South East Asia [7], a strategy that has yet to be introduced by the Indian National Programme.

The results from the present study suggest that following treatment with 20 mg/kg Ambisome, risk factors for VL relapse include male sex, age <5 years and ≥45 years, a slower decrease in splenomegaly during treatment, and a shorter duration of symptoms prior to seeking treatment. It also indicates that when using this regimen the majority of relapses occur 6–12 months post-treatment. Recent evidence from another study in Nepal suggests that a significant number of patients relapse 6–12 months post-treatment with miltefosine [13]. Given the move towards treating VL patients with a single 10 mg/kg dose of Ambisome or short-course combination therapies, and the aim of elimination in the Indian subcontinent, we suggest that a 1-year follow-up is essential and should be recommended for all VL treatments.

## Supporting Information

Checklist S1 STROBE Checklist.
(DOC)
