# Correction: Risk Factors for Visceral Leishmaniasis Relapse in Immunocompetent Patients following Treatment with 20 mg/kg Liposomal Amphotericin B (Ambisome) in Bihar, India

**DOI:** 10.1371/journal.pntd.0002813

**Published:** 2014-04-04

**Authors:** The PLOS Neglected Tropical Diseases Staff

The seventh author’s name is spelled incorrectly. The correct spelling is: Manica Balasegaram.
